# A Visualized Isothermal Amplification Method for Rapid and Specific Detection of Emetic and Non-emetic *Bacillus cereus* in Dairy Products

**DOI:** 10.3389/fmicb.2022.802656

**Published:** 2022-03-28

**Authors:** Lei Wang, Huansen Yang, Kun Wang, Haitao Yang, Mengdi Zhao, Yuping Shang, Fang Wang, Jingquan Dong, Weiguo Zhao, Li Li, Wei Liang, Yan Wang

**Affiliations:** ^1^Department of Central Laboratory, Lianyungang Hospital Affiliated to Jiangsu University, Lianyungang, China; ^2^School of Biotechnology, Jiangsu University of Science and Technology, Zhenjiang, China; ^3^Lianyungang Center for Disease Control and Prevention, Lianyungang, China; ^4^Jiangsu Key Laboratory of Marine Biological Resources and Environment, Jiangsu Key Laboratory of Marine Pharmaceutical Compound Screening, Co-Innovation Center of Jiangsu Marine Bio-industry Technology, Jiangsu Ocean University, Lianyungang, China; ^5^Department of Materials Science and Engineering, Suzhou University of Science and Technology, Suzhou, China; ^6^Laboratory Department of Ningbo First Hospital, Ningbo Hospital of Zhejiang University, Ningbo, China

**Keywords:** *Bacillus cereus*, recombinase polymerase amplification, lateral flow strip, *gyrase B*, cereulide synthetase

## Abstract

*Bacillus cereus* is widely distributed in foods, especially dairy products, and can lead to diarrhea (non-emetic *B. cereus*) and emesis (emetic *B. cereus*). Although diarrhea due to *B*. *cereus* is usually mild, emesis can lead to acute encephalopathy and even death. To develop rapid and sensitive detection methods for *B. cereus* in foods, specific primers targeting the *gyrase B* (*gyrB*) and cereulide synthetase (*ces*) genes were designed and screened using recombinase polymerase amplification (RPA). Probes and base substitutions were introduced to improve specificity and eliminate primer-dependent artifacts. The 5′ ends of the reverse primers and probes were modified with biotin and fluorescein isothiocyanate for detection of RPA products on a lateral flow strip (LFS). The developed RPA-LFS assay allows detection within 20 min at 37°C with no cross-reactivity with other foodborne pathogens. The limit of detection was 10^4^ copies/ml and 10^2^ CFU/ml in pure cultures and milk, respectively. Comparisons with established methods using cream obtained similar results. A specific, rapid, and sensitive RPA-LFS assay was successfully developed for on-site detection of *B. cereus* in dairy products to distinguish emetic from non-emetic strains.

## Highlights

- The present study develops specific, rapid and sensitive RPA-LFS systems to identify Bacillus cereus in dairy products and distinguish non-emetic and emetic *Bacillus cereus* and broadens the diagnosis on site.
- The limit of detection was both 10^4^ copies/mL or 10^2^ CFU/mL in pure cultures and milk. The established methods were also evaluated in cream by comparing with standard culturing method and obtained consistent results.
- The developed RPA-LFS systems allowed detection within 20 min under 37°C with no cross-reaction with other foodborne pathogens.
- Reducing Cross dimer formation and enhancing primer detection performance by introducing base mismatches.

## Introduction

*Bacillus cereus* is a Gram-positive, facultatively anaerobic, rod-shaped, and endospore-forming bacterium. The *B. cereus* group consists of several highly homologous species, including *B. cereus*, *Bacillus thuringiensis*, *Bacillus mycoides*, *Bacillus pseudomycoides*, and *Bacillus cytotoxicus* (Ehling-Schulz and Messelhausser, 2013). *B. cereus*, which has low nutritional requirements and high resistance to complex conditions, is widely distributed in the environment and a common contaminant of dairy products. Usually, sterilization methods, such as high pressure, high temperature, and radiation, kill only live bacteria and have no inactivation effect on spores and some toxins of live bacteria. Spores can stabilize the pasteurization process and rapidly germinate at temperatures of 10–60°C. Toxins are generated by *B*. *cereus* during and after outgrowth as well as during germination (Drobniewski, 1993; Ramarao and Sanchis, 2013). There are two typical clinical symptoms of *B. cereus* toxins: diarrhea, caused by heat-labile enterotoxins, and emesis, caused by heat-stable cereulide (Ulrich et al., 2019). Cereulide is resistant to heat up to 121°C for 90 min and, thus, persists as a toxin in food (Granum and Lund, 1997). Symptoms of diarrhea caused by non-emetic *B. cereus* are usually mild and self-limiting without treatment. However, symptoms of emesis caused by emetic *B. cereus* can lead to acute encephalopathy, liver failure, and even death (Dierick et al., 2005; Rouzeau-Szynalski et al., 2020). Food poisoning usually occurs after eating contaminated dairy products, such as raw milk, cream, and cheese (FAO and WHO, 2014; Porcellato et al., 2018). Pasteurization is insufficient to kill the spores of *B. cereus* (Arnesen et al., 2008). To ensure public health and food safety, rapid and specific methods are needed for the detection and differential diagnosis of emetic and non-emetic *B. cereus* (Yamaguchi et al., 2013).

According to the National Standard of the China (National Food Safety Standard–Food Microbiological Examination–*B. cereus*; GB/T 4789.14-2014), *B. cereus* is identified by culturing, which is both time-consuming and labor-intensive (Anonymous, 2005, 2006). The conventional method for identification of *B*. *cereus* involves dilution of 25 g (or 25 ml) of the sample with 225 ml of diluent and homogenization, followed by serial dilution of the sample homogenate and culturing on nutrient broth plates for 24 to 48 h at 30 ± 1°C. In addition, the accuracy of the results is questionable because of the similar morphologies of bacterial colonies of different *B. cereus* group members (Fricker et al., 2008). Due to the increased frequency of foodborne illnesses, reliable, accurate, and rapid diagnostic methods are urgently needed. With the advances in molecular diagnostic technologies over the past decade, specific genes of *B. cereus* can be detected within a few hours (Wielinga et al., 2011; Dzieciol et al., 2012). Established methods, such as polymerase chain reaction (PCR) and quantitative real-time fluorescence PCR (qPCR), indeed shorten the detection time but require sophisticated thermocycling instruments, which limits applications in low-resource settings. Hence, isothermal amplification techniques are gradually replacing PCR-based methods for on-site detection. Common isothermal amplification techniques include rolling circle amplification, loop-mediated isothermal amplification, and recombinase polymerase amplification (RPA). These methods use specific enzymes for replication of nucleic acids at a constant temperature rather than thermocycling between 55 and 95°C (Gill and Ghaemi, 2008; Craw and Balachandran, 2012; Li and Macdonald, 2015). RPA is a promising method for DNA amplification due to a shorter run time, higher specificity, and greater accuracy as compared to current methods. RPA uses a recombinase to combine single-stranded primers, form nucleoprotein filaments, and recognize complementary sequences in double-stranded template DNA. The amplification step of RPA employs thermostable DNA polymerase with strong strand displacement activity for DNA synthesis at a constant temperature of 37–42°C within 30 min (Li et al., 2019). The amplification products can be detected by traditional agarose gel electrophoresis, which still relies on the availability of lab equipment. Therefore, to further simplify the procedure, a lateral flow strip (LFS) was employed so that the results can be visualized unaided within 10 min. The RPA assay has been applied for detection of various foodborne pathogens (Garrido-Maestu et al., 2017; Yang et al., 2018). Previous studies have reported the use of RPA for detection of *B. cereus* (Liu et al., 2018; Song, 2020). Our group incorporated a base mismatch mechanism in the primer and probe design stage to improve probe detection and avoid false-positive results.

In order to meet the growing demand for fast, reliable, equipment-free technologies for on-site detection of foodborne pathogens, our group established the RPA-LFS assay for detection of the *gyrB* and cereulide synthetase (*ces*) genes of emetic *B*. *cereus* by carefully designing and modifying specific primers and probes, and optimizing the reaction conditions. The detection sensitivity and specificity of the RPA-LFS assay were compared to those of standard culturing methods for the detection of emetic *B*. *cereus* in raw milk and cream. The proposed RPA-LFS assay significantly improves on-site detection of emetic and non-emetic *B. cereus* in dairy products.

## Materials and Methods

### Bacterial Strains and Dairy Products

Reference strains of emetic and non-emetic *B. cereus* (National Collection of Type Cultures [NCTC] 11143 and American Type Culture Collection [ATCC] 14579, respectively) were used to establish the RPA-LFS assay. Ten sputum-isolated strains of non-emetic *B. cereus* and nine sputum-isolated strains of emetic *B. cereus* and other *B. cereus* group members, including *B. thuringiensis* (ATCC 10792), *B. cytotoxicus* (Marine Culture Collection of China [MCCC] 1A12730), *B. mycoides* (ATCC 6462), and *B. pseudomycoides* (MCCC 1A12732), were selected to evaluate the specificity of the RPA-LGFS assay. *Bacillus* strains (*Bacillus amyloliquefaciens* [ATCC 23842], *Bacillus subtilis* [ATCC 6051], *Bacillus brevis* [MCCC 1A06695], and *Bacillus licheniformis* [ATCC 14580]) and several common foodborne pathogens (*Listeria monocytogenes* [ATCC 19115], *Salmonella enteritidis* [ATCC 14028], *Staphylococcus aureus* [ATCC 6538], and *Escherichia coli* O157 [ATCC 43888]) were used to assess the exclusivity of the assay.

The dairy products (i.e., raw milk and cream) used in the present study were obtained from a local market. The presence of *B. cereus* was confirmed in accordance with the National Food Safety Standard GB/T 4789.14-2014. The raw milk and cream were free of *B. cereus*, indicating that the detection of *B*. *cereus* was due to external addition. Spiked raw milk was used to determine the sensitivity of the RPA-LFS assay. The cream was used to detect contamination with emetic and non-emetic *B. cereus* bacteria and spores, and to compare the detection results of the RPA-LFS assay with those of standard culturing methods.

The 50 clinical samples used in this study were all from the Lianyungang Center for Disease Control and Prevention, including commercial dairy and expired dairy (which may contain *B. cereus* contamination). RPA-LFS system and qPCR were used for detection, respectively.

### Preparation of Spore Suspensions

Spore suspensions of emetic and non-emetic *B. cereus* were prepared as reported previously and stored at 4°C (Frentzel et al., 2016). Spore purity and the absence of vegetative bacteria were verified by light microscopy. To measure the spore concentrations, 1 ml of spore suspension was diluted with 10 ml of phosphate-buffered saline (PBS) and spread on nutrient agar plates, which were incubated for 24 h at 30°C. The bacterial counts of the plates ranged from 20 to 200 colony-forming units (CFU).

### Preparation of Genomic DNA From Pure Cultures and Dairy Products

The genomic DNA (gDNA) of emetic and non-emetic *B*. *cereus* in pure cultures and dairy products was extracted using the TIANamp Genomic DNA Kit (Tiangen Biotech Co., Ltd., Beijing, China) in accordance with the manufacturer’s instructions. In dairy products, gDNA extraction from vegetative bacteria included enzymatic digestion of milk proteins as described by Dzieciol et al. (2012) prior to extraction using the TIANamp Genomic DNA Kit. To evaluate the RPA-LFS assay for detection of spore suspensions in spiked cream samples, gDNA was extracted from vegetative bacteria and spores as described by Dzieciol et al. (2012). The extraction method included enzymatic digestion of milk proteins and spores and mechanical breakage of vegetative bacteria and spores before extraction of gDNA using the TIANamp Genomic DNA Kit. The concentration and purity of the extracted gDNA from pure cultures and dairy products were measured using a Qubit 4 fluorometer (Thermo Fisher Scientific, Waltham, MA, United States). If not specified, 1 μl of gDNA at 10^6^ CFU/ml was used as a template in the reactions.

### Primer and Probe Design

Primers and probes targeting the *gyrB* gene (GenBank accession number NC_004722.1) and *ces* gene (GenBank accession number DQ360825.1) were designed using Primer Premier 5.0 software (PREMIER Biosoft, Palo Alto, CA, United States). The 5′ ends of the reverse primers and probes were labeled with biotin and fluorescein isothiocyanate (FITC), respectively. The 3′ ends of the probes were blocked with a C3 spacer. The base located more than 30 bases away from the 5′ end and 15 bases away from the 3′ end was replaced with a tetrahydrofuran site. The primers and probes targeting the *gyrB* gene were used to specifically detect *B. cereus* group members among different foodborne pathogens, and the *ces* gene was used to identify emetic *B. cereus* among *B. cereus* group members. All primers and probes were synthesized by General Biosystems (Anhui) Co., Ltd. (Anhui, China).

### Recombinase Polymerase Amplification Assay

The RPA assay was performed in accordance with the manufacturer’s instructions of the TwistAmp^®^ Liquid DNA Amplification Kit (TwistDx Inc., Maidenhead, United Kingdom). Each RPA reaction contained 25 μl of 2 × reaction buffer, 5 μl of 10 × Basic e-mix, 2.5 μl of 20 × core mix, 2.1 μl of each primer (10 μM), 9.8 μl of distilled water, and 1 μl of the template. To initiate the reaction, 2.5 μl of magnesium acetate (280 mM) was added to the mixture. After protein-induced DNA bending and centrifugation, the mixture was immediately incubated for 30 min at 37°C. The amplification products were purified with the MonPure™ Gel and PCR Clean Kit (Monad Biotech Co., Ltd., Wuhan, China) and separated by electrophoresis on a 1.5% agarose gel.

### Recombinase Polymerase Amplification-Lateral Flow Strip Assay

The RPA assay was performed according to the manufacturer’s instructions of the TwistAmp^®^ DNA Amplification nfo Kit. The RPA reaction contained 29.5 μl of rehydration buffer, 2.1 μl of forward primer (10 μM), 2.1 μl of reverse primer (10 μM), 0.6 μl of probe (10 μM), 1 μl of template, a dried enzyme pellet, and distilled water (up to 47.5 μl). To initiate the reaction, 2.5 μl of magnesium acetate (280 mM) was added to the mixture. After fully bending and brief centrifugation, the mixture was immediately incubated for 5–30 min at 30°C–42°C. Then, 5 μl of the amplification products was used for detection with an LFS (Ustar Biotechnologies Ltd., Hangzhou, China). The RPA products were added to the sample pad of LFS, and the stick of the LFS was inserted into 100 μl of the sample buffer for 3 min and then visually read.

### Specificity of the Recombinase Polymerase Amplification-Lateral Flow Strip Assay for Emetic and Non-emetic *Bacillus cereus*

To evaluate the specificity of the RPA-LFS assay, the gDNA of *B. cereus* group members (emetic and non-emetic *B. cereus*, *B. cytotoxicus*, *B. thuringiensis*, *B. mycoides*, *B. pseudomycoides*) was extracted and then tested using the optimized RPA-LFS conditions. The tested samples included ten *B. cereus* sputum-isolated strains, nine emetic *B. cereus* sputum-isolated strains, and one standard strain of each bacterium. Each experiment was performed in three triplicates.

### Sensitivity of the Recombinase Polymerase Amplification-Lateral Flow Strip Assay With Pure Cultures, Spiked Milk, and Mixtures With Other Foodborne Pathogens

Raw milk was obtained from a local market and verified as *B. cereus*-free in accordance with National Food Safety Standard (GB/T 4789.14-2014). To assess the limit of detection (LOD) of the RPA-LFS assay for emetic and non-emetic *B. cereus*, gDNA extracted from emetic and non-emetic *B*. *cereus* was mixed with 10^8^ copies/ml of *L. monocytogenes* gDNA and raw milk spiked with 10-fold dilutions of gDNA of emetic and non-emetic *B*. *cereus* (final concentration of 10^3^–10^8^ copies/ml or 10–10^6^ CFU/ml, respectively). Each experiment was performed in triplicate.

### Detection of Emetic and Non-emetic *Bacillus cereus* in Spiked Cream

Cream was purchased from a local market and verified as *B. cereus*-free in accordance with the National Food Safety Standard GB/T 4789.14-2014 to establish artificially spiked emetic and non-emetic *B*. *cereus* models. To evaluate the lower LOD of vegetative bacteria, 25 g of cream was dissolved in 225 ml of nutrient broth, incubated with either emetic or non-emetic *B. cereus* to final concentrations of 10^0^–10^2^ CFU/ml, and then enriched at 30°C. An equal amount PBS was used as a control. Then, 1 ml of the enrichment solution was collected at 0, 2, 4, and 6 h and gDNA was extracted from bacterial cultures. The RPA-LFS assay was performed as described above. Cell counts of the contaminated food samples at each time point were determined on nutrient agar after incubation for 24 h at 30°C. For spore evaluation, 25 g of cream was dissolved in 225 ml of nutrient broth and incubated with spores of either emetic or non-emetic *B. cereus* to final concentrations of 10^3^–10^5^ CFU/ml. An equal amount PBS was used as a control. Then, 1 ml of the contaminated samples was collected and gDNA was extracted from the vegetative bacteria and spores. Cell counts of the contaminated samples were also determined. Each experiment was performed in three triplicates.

### TaqMan qPCR

The primers and probes used in this study are listed in Table 1. The primers and probes for qPCR were designed to specifically target the *gyrB* gene of *B. cereus* (Dzieciol et al., 2012) and the *ces* gene of emetic *B. cereus*. The plasmid pUC19 was used as an internal amplification control (IAC). Primers and probes were also designed to specifically target the pMB1 replicon *rep* (Fricker et al., 2007). The TaqMan qPCR reaction mixture contained 12.5 μl of MonAmp™ TaqMan qPCR Mix, 0.5 μM of forward and reverse primers, 0.2 μM of probe, 1 μl of gDNA, 170 copies of plasmid DNA pUC19, and distilled water to a final volume of 25 μl. The primers and probes specifically targeted the *gyrB* or *ces* gene and IAC. The cycling conditions consisted of an initial denaturation step at 95°C for 10 min, followed by 40 cycles at 95°C for 15 s and 55°C for 60 s. qPCR was conducted using a LightCycler^®^ 480 Instrument (Roche Diagnostics GmbH, Mannheim, Germany). The results were analyzed using LightCycler^®^ 480 Instrument Software version 1.1.1 in accordance with the Minimum Information for Publication of qPCR Experiments guidelines (Bustin et al., 2009). Each experiment was performed in three duplicates.

**TABLE 1:** Primers and probes designed for emetic and non-emetic *Bacillus cereus* RPA-LFS detection systems.

Primers/Probes	Primer sequences	Size (bp)	References
*gyrB*-F	TACATCGTGAAGGTA AAATCCATTACCAAA AATAC	32	This study
*gyrB*-R	Biotin-TTATATTGAAGAGAA ACCTCAACCTGAATACCA	34	
*gyrB*-P	FITC-TTAAAAGTCATTGGTGACACCGATCAAACA[THF]GAGCA ATAACTCGAT–/C3-spacer/	46	
*ces*-F	ATAGTGGGAA AATAACGAGA AATGCATTTC	30	
*ces*-P	FITC-TCAAA AACAGTTTGAGAACGGGGCATATAGAG [THF]GATTACACAAAAGAT-C3-spacer	48	
*ces*-P-m	FITC-TCAAA AACAGTTTGAGAACGGGTCATCTAGAG[THF]GATTACACAAGAGAT-C3-spacer	48	
*ces*-R	Biotin-GGGAGGATACGCTTTTGCCCAACTTACTTT	30	
*ces*-R-m	Biotin-GGGAGGATACGCTTATGCCCTACTTACATT	30	
q*gyrB*-F	GCCCTGGTATGTATATTGGATCTAC	25	Dzieciol et al., 2012
q*gyrB*-P	FAM-CCATTTTTTCTTGTATACCAACT-MGB	23	
q*gyrB*-R	GGTCATAATAACTTCTACAGCAGGA	25	
q*ces*-F	CGCCGAAAGTGATTATACCAA	21	Fricker et al., 2007
q*ces*-P	CY5-GGGAAAATAACGAGAAATGCA-BHQ2	21	
q*ces*-R	TATGCCCCGTTCTCAAACTG	20	
qIAC-F	GCAGCCACTGGTAACAGGAT	20	
qIAC-P	HEX-AGAGCGAGGTATGTAGGCGG-TAMRA	20	
qIAC-R	GCAGAGCGCAGATACCAAAT	20	

*F, forward primer; R, reverse primer; P, probe; THF, tetrahydrofuran.*

## Results

### Performance of the *gyrB* Primers and Probes

**FIGURE 1:**
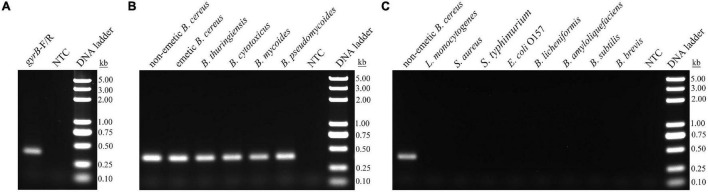
Sensitivity of the primers for *gyrB* by RPA amplification. **(A)** Performance of the *gyrB* primers using the gDNA of non-emetic *Bacillus cereus* as a template. **(B)** Performance of the *gyrB* primers by inclusivity testing. **(C)** Performance of the *gyrB* primers by exclusivity testing. The gDNA of bacterial strains used in each RPA reaction is indicated on the top of each lane. NTC, no template control. The size of the DNA ladder is indicated beside each band.

All strains were verified by detection of the *gyrB* and *ces* genes (Table 2). Meanwhile, the IAC was detected in all strains with a mean *Cq* value of 31.67 (*SD* < 0.5). A pair of specific primers targeting the conserved regions of the *gyrB* gene was designed, and performance was tested in the RPA reactions using the gDNA of non-emetic *B. cereu*s. The purified RPA products were separated by electrophoresis on a 1.5% agarose gel. A 379-bp band was obtained, and no obvious primer–dimer or non-specific amplification was observed in the no template control (NTC) (Figure 1A). To screen the specificity of the primers for *gyrB*, the RPA assay was conducted using the gDNA of the *B. cereus* group members (emetic and non-emetic *B. cereus*, *B. thuringiensis*, *B. cytotoxicus*, *B. mycoides*, and *B. pseudomycoides*), other *Bacillus* strains (*B*. *amyloliquefaciens*, *B*. *licheniformis*, *B*. *brevis*, and *B*. *subtilis*), and common foodborne pathogens (*L*. *monocytogenes*, *S*. *aureus*, *S*. *enteritidis*, and *E*. *coli* O157). The results indicated that the bands of the *B. cereus* group members were the expected size, thereby confirming the specificity of the *gyrB* primers (Figure 1B) with no detection of the other bacteria (Figure 1C).

**TABLE 2:** Amplification of *gyrB* and *ces* gene in different bacterial strains by the TaqMan qPCR method.

Taxon	Number	Cq^d^ *value of *gyrB**	Cq^d^ *value of *ces**
Non-emetic *Bacillus cereus*	ATCC^a^ 14579	22.17	No *Cq*
Non-emetic *Bacillus cereus*	Sputum isolated strain #1	22.12	No *Cq*
Non-emetic *Bacillus cereus*	Sputum isolated strain #2	23.18	No *Cq*
Non-emetic *Bacillus cereus*	Sputum isolated strain #3	21.78	No *Cq*
Non-emetic *Bacillus cereus*	Sputum isolated strain #4	22.67	No *Cq*
Non-emetic *Bacillus cereus*	Sputum isolated strain #5	22.12	No *Cq*
Non-emetic *Bacillus cereus*	Sputum isolated strain #6	22.19	No *Cq*
Non-emetic *Bacillus cereus*	Sputum isolated strain #7	23.07	No *Cq*
Non-emetic *Bacillus cereus*	Sputum isolated strain #8	22.22	No *Cq*
Non-emetic *Bacillus cereus*	Sputum isolated strain #9	23.15	No *Cq*
Non-emetic *Bacillus cereus*	Sputum isolated strain #10	22.18	No *Cq*
Emetic *B. cereus*	NCTC^b^ 11143	23.42	23.17
Emetic *B. cereus*	Sputum isolated strain #1	21.45	21.45
Emetic *B. cereus*	Sputum isolated strain #2	22.67	23.87
Emetic *B. cereus*	Sputum isolated strain #3	23.89	22.99
Emetic *B. cereus*	Sputum isolated strain #4	21.65	21.23
Emetic *B. cereus*	Sputum isolated strain #5	22.44	23,65
Emetic *B. cereus*	Sputum isolated strain #6	22.13	22.18
Emetic *B. cereus*	Sputum isolated strain #7	21.76	23.19
Emetic *B. cereus*	Sputum isolated strain #8	21.98	23.18
Emetic *B. cereus*	Sputum isolated strain #9	22.16	23.11
*B. thuringiensis*	ATCC 10792	21.87	No *Cq*
*B. cytotoxicus*	MCCC^c^ 1A12730	22.65	No *Cq*
*B. mycoides*	ATCC 6462	23.44	No *Cq*
*B. pseudomycoides*	MCCC 1A12732	24.01	No *Cq*
*B. amyloliquefaciens*	ATCC 23842	No *Cq*	No *Cq*
*B. subtilis*	ATCC 6051	No *Cq*	No *Cq*
*B. brevis*	MCCC 1A06695	No *Cq*	No *Cq*
*B. licheniformis*	ATCC 14580	No *Cq*	No *Cq*
*Listeria monocytogene*	ATCC 19115	No *Cq*	No *Cq*
*Salmonella enteritidis*	ATCC 14028	No *Cq*	No *Cq*
*Staphylococcus aureus*	ATCC 6538	No *Cq*	No *Cq*
*Escherichia coli* O157	ATCC 43888	No *Cq*	No *Cq*

*^a^ATCC, Manassas, VA, United Staes.*

*^b^NCTC, National Collection of Type Cultures, United Kingdom.*

*^c^MCCC, Marine Culture Collection of China.*

*^d^Cq is cycle number measured at the cross point between the amplification curve and the fluorescence threshold.*

**FIGURE 2:**
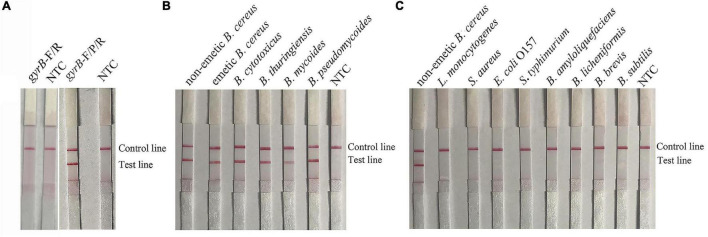
Sensitivity of the primers and probes for *gyrB* for the RPA-LFS assay. **(A)** Performance of the *gyrB* primers and probe using the gDNA of non-emetic *B. cereus* as a template. **(B)** Performance of the *gyrB* primers and probe by inclusivity testing. **(C)** Performance of the *gyrB* primers and probe by exclusivity testing. The gDNA of bacterial strains used in each RPA reaction is indicated on the top of each lane. NTC, no template control.

Although the RPA-LFS assay is sensitive, it was difficult to distinguish between the target gDNA and primer–dimers on the LFS. Thus, specific probes for the RPA reactions were designed to improve the specificity and eliminate primer-dependent artifacts (Piepenburg et al., 2006). A probe specific for *gyrB*-P was designed based on the forward and reverse primers for *gyrB*. After RPA amplification with *gyrB*-F/R and *gyrB*-F/R/P, samples were added to the sample pad. The results showed that the specific primers and probes obtained bands of expected sizes with no signal for the NTC (Figure 2A). Thus, the primers and probe for *gyrB*-F/R/P were used to further assess the specificity of the RPA-LFS assay for *B. cereus*. The results showed that bands of expected sizes were obtained for all *B. cereus* group members (Figure 2B). The RPA-LFS assay was also verified with six non-emetic *B. cereus* and nine emetic *B. cereus* isolates from foods (data not shown). No bands were detected for the other *Bacillus* strains and common foodborne pathogens (Figure 2C).

### Screening the Cereulide Synthetase Primers and Probes of Emetic *Bacillus cereus*

**FIGURE 3:**
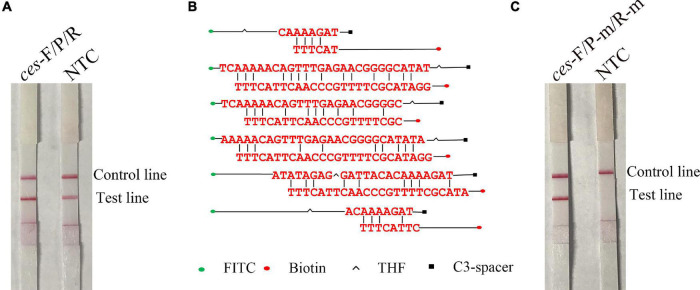
Specific and mismatched reverse primer/probes targeting the *ces* gene and performance in identifying emetic *B. cereus*. **(A)** Performance of *ces*-F/R/P using RPA-LFS. **(B)** Possible dimer formation between the specific reverse primers for *ces*-R and probe for *ces*-P. **(C)** Performance of *ces*-F/R-m/P-m using the RPA-LFS assay. NTC, no template control.

To further determine whether the *B. cereus* is emetic or not, a pair of primers specific for *ces*-F/R and a probe for *ces*-P were designed to amplify the *ces* gene in the RPA reaction with the gDNA of emetic *B. cereus* as a template and detected on an LFS and agarose gel. Positive signals were obtained for the tested samples as well as the NTC (Figure 3A and Supplementary Figure 1A). Thus, the possibility of false-positive results was explored by targeting the base pair common to the reverse primer and probe (Figure 3B). Next, base substitutions were introduced and detected using the modified reverse primer and probe for *ces*-F/R-m/P-m to test the performance of the RPA-LFS assay. The results indicated that the modified reverse primer and probe specifically amplified the target gene using the gDNA of emetic *B. cereus* with no false-positive signal (Figure 3C and Supplementary Figure 1B).

**FIGURE 4:**
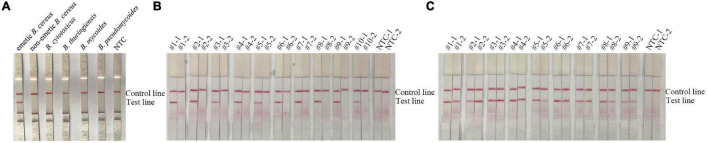
Sensitivity of the RPA-LFS assay for detection of *B. cereus*. **(A)** Exclusivity testing of the RPA-LFS assay for detection of emetic *B. cereus* RPA-LFS. The gDNA of bacterial strains used in each RPA reaction is indicated on the top of each lane. NTC, no template control. **(B)** Exclusivity testing of ten sputum-isolated strains of non-emetic *B. cereus* with the RPA-LFS assay. The primers and probe on the left of each group were *gyrB* and *ces*-F/R-m/P-m on the right. NTC, no template control. **(C)** Exclusivity testing of nine sputum-isolated strains of emetic *B. cereus* with the RPA-LFS assay. The primers and probe on the left of each group were *gyrB* and *ces*-F/R-m/P-m on the right. NTC, no template control.

The specificity of the RPA-LFS assay was further evaluated using other *B. cereus* group members as templates. The results showed that only emetic *B. cereus* produced a positive signal (Figure 4A and Supplementary Figure 1C). The specificity of the RPA-LFS assay was also tested using ten non-emetic *B. cereus* and nine emetic *B. cereus* isolates from foods (Figures 4B,C). The results showed that the RPA-LFS assay specifically recognized only emetic *B. cereus* among the *B. cereus* group members.

### Optimization of the Recombinase Polymerase Amplification-Lateral Flow Strip Assay for Detection of Emetic and Non-emetic *Bacillus cereus*

**FIGURE 5:**
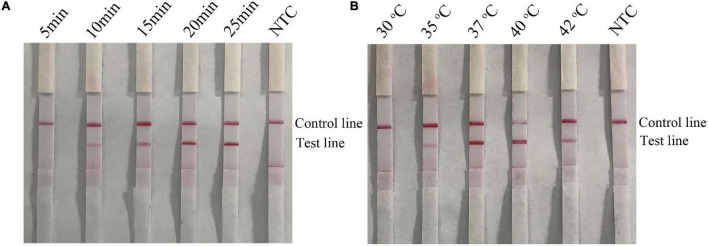
Screening of optimal time and temperature parameters for the *B. cereus* RPA-LFS assay. **(A)** The performance of the *B. cereus* RPA-LFS assay at different times (5–25 min). **(B)** The performance of the *B. cereus* RPA-LFS assay at different temperatures (30–42°C). The detailed time and temperature are indicated on the top of each lane. NTC, no template control.

**FIGURE 6:**
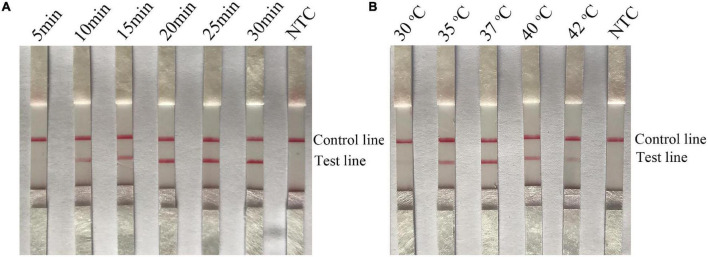
Screening of the optimal time and temperature parameters for the emetic *B. cereus* RPA-LFS assay. **(A)** The performance of the emetic *B. cereus* RPA-LFS assay at different times (5–30 min). **(B)** The performance of the emetic *B. cereus* RPA-LFS assay at different temperatures (30–42°C). The detailed time and temperature are indicated on the top of each lane. NTC, no template control.

The parameters of time and temperature were optimized to improve the performance of the RPA-LFS assay. The time was adjusted to 5–25 min for *B. cereus* and 5–30 min for emetic *B. cereus*. As shown in Figure 5A, pink test lines appeared at 10 min and became clearer from 15 to 25 min. There were no significant differences in the intensities of lines appearing at 20 and 25 min. In Figure 6A, positive signals also appeared at 10 min and became more obvious from 15 to 30 min. There were no significant differences in the intensities of the lines appearing at 20 and 30 min. Thus, a reaction time of 20 min was finally selected for the RPA-LFS assay for detection of both emetic and non-emetic *B. cereus*. The temperature was adjusted from 30 to 42°C, while the reaction time was set at 20 min. As shown in Figures 5B, 6B, signals appeared on the test lines at 35, 37, 40, and 42°C and were most obvious at 37°C. The time and temperature parameters were tested using three duplicates. Hence, the optimal conditions for the RPA-LFS assay for detection of emetic and non-emetic *B. cereus* were 37°C for 20 min.

### Sensitivity of the Recombinase Polymerase Amplification-Lateral Flow Strip Assay for *Bacillus cereus* Detection in Pure Cultures, Spiked Milk, and Mixtures With Other Foodborne Pathogens

**FIGURE 7:**
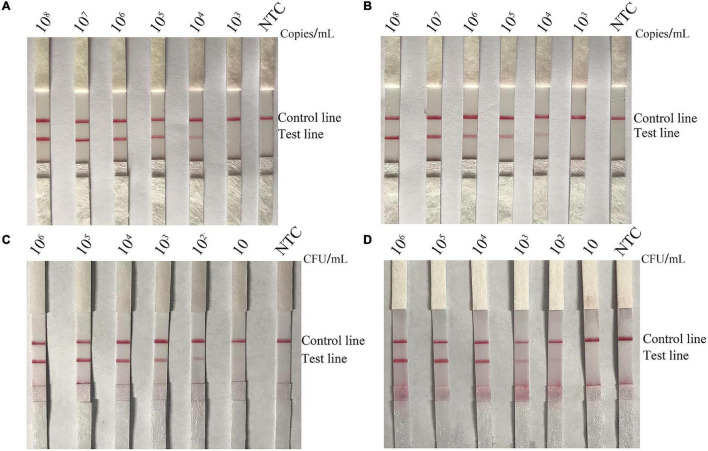
Screening of the detection sensitivity of the non-emetic *B. cereus* RPA-LFS assay. To explore the minimum detection limit of the *B*. *cereus* RPA-LFS assay, 10-fold dilutions of **(A)** non-emetic *B. cereus* gDNA (10^8^–10^3^ copies/ml), **(B)** non-emetic *B. cereus* gDNA (10^8^–10^3^ copies/ml) mixed with *L. monocytogenes* gDNA to a concentration of 10^8^ copies/ml, **(C)** *B. cereus* gDNA (10^6^–10 CFU/ml), and **(D)** *B. cereus* spiked raw milk (10^6^–10 CFU/ml) were used as templates. The detailed template amounts are indicated on the top of each lane. NTC, no template control.

**FIGURE 8:**
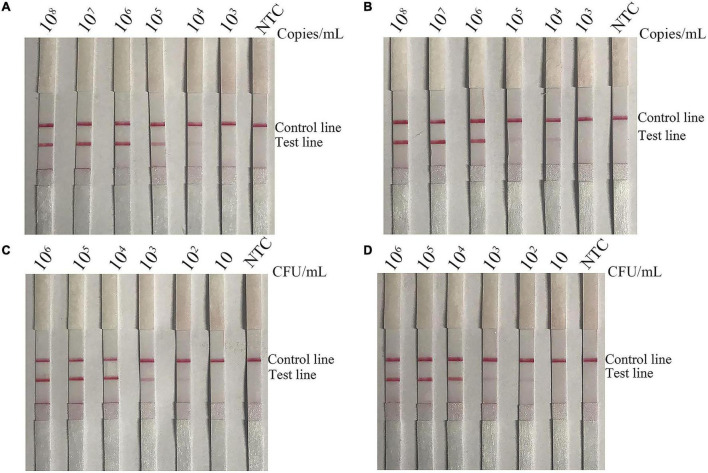
Screening of the detection sensitivity the emetic *B. cereus* RPA-LFS assay. To explore the minimum detection limit of the emetic *B*. *cereus* RPA-LFS assay, 10-fold dilutions of **(A)** emetic *B. cereus* gDNA (10^8^–10^3^ copies/ml), **(B)** emetic *B. cereus* gDNA (10^8^–10^3^ copies/ml) mixed with *L. monocytogenes* gDNA to a concentration of 10^8^ copies/ml, **(C)** emetic *B. cereus* gDNA (10^6^–10 CFU/ml), and **(D)** emetic *B. cereus* spiked raw milk (10^6^–10 CFU/ml) were used as templates. The detailed template amounts are indicated on the top of each lane. NTC, no template control.

To evaluate the sensitivity of the RPA-LFS assay for detection of emetic and non-emetic *B. cereus*, 10-fold dilutions of emetic and non-emetic *B*. *cereus* of 10^3^–10^8^ copies/ml and 10–10^6^∼10 CFU/ml were utilized as template DNA. The sensitivity results showed that the minimum amount of gDNA was 10^4^ copies/ml or 10^2^ CFU/ml (Figures 7A,C, 8A,C). To determine whether contamination with other strains would interfere with detection, gDNA of *L. monocytogenes* was mixed with 10-fold dilutions of gDNA of emetic and non-emetic *B*. *cereus* (10^3^–10^8^ copies/ml) to a concentration of 10^8^ copies/ml, and the mixed gDNA was detected with the RPA-LFS assay for emetic and non-emetic *B. cereus*. The results were the same as those obtained with the purified gDNA (Figures 7B, 8B). Raw milk was spiked with inactivated emetic and non-emetic *B*. *cereus* to a final concentration of 10–10^6^ 10 CFU/ml. gDNA was extracted to evaluate the sensitivity of the RPA-LFS assay for detection of emetic and non-emetic *B. cereus*. The results indicated that the minimum amount of emetic and non-emetic *B. cereus* in spiked raw milk was 10^2^ CFU/ml, which was the same sensitivity as with the purified gDNA (Figures 7D, 8D).

### Performance of Recombinase Polymerase Amplification-Lateral Flow Strip Assay for Detection of Emetic and Non-emetic *Bacillus cereus* in Artificially Contaminated Cream Samples

To further evaluate the performance of the RPA-LFS assay, cream was contaminated with different amounts of emetic and non-emetic *B. cereus* cultures and spore suspensions. In order to evaluate the ability of the RPA-LFS assay to detect vegetative bacteria in spiked cream, gDNA was extracted from cream samples spiked with 10^0^–10^2^ CFU/ml of emetic and non-emetic *B*. *cereus* cultures. Without enrichment, the RPA-LFS assay could only detect gDNA in cream samples spiked with 10^2^ CFU/ml, but not 10 CFU/ml. After enrichment for 2 h, the RPA-LFS assay was able to detect gDNA in cream samples spiked with concentrations of only 1 CFU/ml. After enrichment for 4 h, the RPA-LFS assay detected gDNA in all spiked samples (Table 3). To mimic natural contamination, cream was spiked with spores of emetic and non-emetic *B*. *cereus*. Total gDNA of vegetative bacteria and spores was extracted. Meanwhile, the cell counts were determined by standard culturing. Consistent results were obtained with both methods (Table 4).

**TABLE 3:** RPA-LFS detection of *Bacillus cereus* in artificial contaminated cream samples with emetic and non-emetic *B*. *cereus.*

Contaminated concentrations (CFU/mL)	Detection methods	Contaminated samples	Enrichment time (h)			
			**0**	**2**	**4**	**6**
10^0^	RPA-LFS for *gyrB*	Non-emetic *Bacillus cereus*	–	–	+	+
		Emetic *B. cereus*	–	–	+	+
		NC	–			
		NTC	–			
	RPA-LFS for *ces*	Non-emetic *Bacillus cereus*	–	–	–	–
		Emetic *B. cereus*	–	–	+	+
		NC	–			
		NTC	–			
	GB/T 4789.14-2014	Non-emetic *Bacillus cereus*	0	7.0 × 10	2.0 × 10^3^	5.5 × 10^5^
		Emetic *B. cereus*	0	5.5 × 10	8.7 × 10^2^	1.1 × 10^5^
		NC	0			
10	RPA-LFS for *gyrB*	Non-emetic *Bacillus cereus*	–	+	+	+
		Emetic *B. cereus*	–	+	+	+
		NC	–			
		NTC	–			
	RPA-LFS for *ces*	Non-emetic *Bacillus cereus*	–	–	–	–
		Emetic *B. cereus*	–	+	+	+
		NC	–			
		NTC	–			
	GB/T 4789.14-2014	Non-emetic *Bacillus cereus*	1.6 × 10	2.3 × 10^2^	1.0 × 10^4^	4.5 × 10^6^
		Emetic *B. cereus*	1.8 × 10	1.1 × 10^2^	6.0 × 10^3^	6.0 × 10^5^
		NC	0			
10^2^	RPA-LFS for *gyrB*	Non-emetic *Bacillus cereus*	+	+	+	+
		Emetic *B. cereus*	+	+	+	+
		NC	–			
		NTC	–			
	RPA-LFS for *ces*	Non-emetic *Bacillus cereus*	–	–	–	–
		Emetic *B. cereus*	+	+	+	+
		NC	–			
		NTC	–			
	GB/T 4789.14-2014	Non-emetic *Bacillus cereus*	2.3 × 10^2^	2.5 × 10^3^	3.8 × 10^5^	9.5 × 10^7^
		Emetic *B. cereus*	2.1 × 10^2^	1.4 × 10^3^	1.4 × 10^5^	2.1 × 10^7^
		NC	0			

*+, positive results; –, negative results; NC, negative control; NTC, no template control.*

**TABLE 4:** Identification of *Bacillus cereus* in artificial contaminated cream samples with the non-emetic *B. cereus* spores and emetic *B. cereus* spores.

Spore concentrations (CFU/mL)	Non-emetic *B. cereus* spore spiked cream			Emetic *B. cereus* spore spiked cream		
	**RPA-LFS for *gyrB***	**RPA-LFS for *ces***	**GB/T 4789.14-2014**	**RPA-LFS for *gyrB***	**RPA-LFS for *ces***	**GB/T 4789.14-2014**
10^5^	+	–	8.3 × 10^5^	+	+	3.7 × 10^5^
10^4^	+	–	9.5 × 10^4^	+	+	3.1 × 10^4^
10^3^	+	–	1.3 × 10^4^	+	+	2.2 × 10^3^
NC	–	–	0	–	–	0
NTC	–	–	/	–	–	/

*+, positive results; –, negative results; /, no involvement; NC, negative control; NTC, no template control.*

### Results for Clinical Samples

The RPA-LFS system was applied to detect *B. cereus* in potentially contaminated commercial dairy and expired dairy samples, and the results were compared with qPCR methods. In this study, 50 clinical samples were collected and seven were detected with *B. cereus* contamination, two of which were contaminated with emetic *B. cereus*. These samples were also tested by qPCR, and we considered a *Ct* value < 32 as positive contamination in the qPCR results. Five non-emetic and two emetic *B. cereus* were successfully detected, and the results of the RPA-LFS system were consistent with the results of qPCR (Table 5).

**TABLE 5:** Detection performance of the RPA-LFS system and quantitative PCR.

No.	RPA-LFS		Quantitative PCR *Ct* value (*n* = 3)		No.	RPA-LFS		Quantitative PCR *Ct* value (*n* = 3)		No.	RPA-LFS		Quantitative PCR *Ct* value (*n* = 3)	
								
	*gyrB*	*ces*	*gyrB*	*ces*		*gyrB*	*ces*	*gyrB*	*ces*		*gyrB*	*ces*	*gyrB*	*ces*
1	–	–	36.32 ± 0.19	36.32 ± 0.37	18	–	–	33.13 ± 0.32	34.17 ± 0.21	35	–	–	33.62 ± 0.34	35.02 ± 0.18
2	–	–	34.22 ± 0.24	36.17 ± 0.33	19	–	–	35.35 ± 0.35	32.12 ± 0.39	36	–	–	39.02 ± 0.23	34.36 ± 0.26
3	–	–	38.31 ± 0.19	36.72 ± 0.11	20	–	–	33.32 ± 0.47	32.99 ± 0.03	37	–	–	35.32 ± 0.22	34.54 ± 0.33
4	–	–	35.38 ± 0.39	33.34 ± 0.42	21	–	–	35.02 ± 0.42	35.33 ± 0.28	38	–	–	36.31 ± 0.11	35.62 ± 0.14
5	–	–	32.46 ± 0.23	33.18 ± 0.45	22	+	–	24.36 ± 0.27	35.29 ± 0.22	39	–	–	35.02 ± 0.23	35.98 ± 0.35
6	+	–	22.12 ± 0.35	35.33 ± 0.41	23	–	–	35.13 ± 0.23	36.44 ± 0.17	40	–	–	34.72 ± 0.45	33.02 ± 0.24
7	–	–	35.23 ± 0.34	34.26 ± 0.38	24	–	–	36.56 ± 0.32	37.13 ± 0.34	41	+	+	23.02 ± 0.15	21.42 ± 0.32
8	–	–	36.38 ± 0.27	37.83 ± 0.36	25	–	–	37.23 ± 0.19	35.02 ± 0.17	42	+	–	22.42 ± 0.36	33.52 ± 0.35
9	–	–	36.24 ± 0.49	35.76 ± 0.23	26	–	–	35.52 ± 0.39	38.39 ± 0.20	43	–	–	37.76 ± 0.43	38.39 ± 0.32
10	+	+	23.02 ± 0.32	22.17 ± 0.23	27	–	–	33.62 ± 0.43	35.25 ± 0.48	44	–	–	36.23 ± 0.23	34.77 ± 0.36
11	–	–	35.12 ± 0.31	37.35 ± 0.34	28	–	–	35.34 ± 0.34	36.32 ± 0.19	45	–	–	35.02 ± 0.45	37.45 ± 0.34
12	–	–	35.72 ± 0.41	35.33 ± 0.23	29	–	–	37.17 ± 0.34	35.45 ± 0.43	46	–	–	37.02 ± 0.35	36.36 ± 0.44
13	–	–	34.32 ± 0.36	33.32 ± 0.39	30	–	–	34.48 ± 0.24	37.31 ± 0.13	47	–	–	35.35 ± 0.23	37.36 ± 0.33
14	+	–	22.32 ± 0.12	33.32 ± 0.17	31	+	–	22.34 ± 0.35	39.22 ± 0.14	48	–	–	36.42 ± 0.41	38.35 ± 0.39
15	–	–	37.32 ± 0.29	38.34 ± 0.49	32	–	–	35.02 ± 0.22	34.56 ± 0.31	49	–	–	36.39 ± 0.37	35.02 ± 0.38
16	–	–	35.02 ± 0.24	36.52 ± 0.33	33	–	–	37.34 ± 0.29	34.34 ± 0.22	50	–	–	34.52 ± 0.13	36.28 ± 0.27
17	–	–	34.51 ± 0.25	35.32 ± 0.30	34	–	–	34.14 ± 0.23	34.25 ± 0.22					

*“+” and Ct value < 32 mean positive. “–” and Ct value > 32 mean negative.*

## Discussion

Food safety, which is threatened by various pathogens, continues to attract public attention. Hence, there is a need to rapidly detect foodborne pathogenic bacteria to avoid with food-poisoning outbreaks. *B. cereus* is an endospore-forming pathogenic bacterium with extreme survival ability that is associated with a wide range of foodborne diseases, especially in foods with high protein levels, such as dairy products (Goepfert et al., 1972).

Several methods have been developed for the detection of *B. cereus* and its pathogenic factors (Ramarao et al., 2020). For example, Manpreet et al. (2022) developed a portable colorimetric point-of-care device for the detection of *B. cereus* in food samples with a detection range of 10^2^–10^3^ CFU/ml, while Zheng et al. (2022) developed a cDNA-based up-conversion fluorescence spectrum copy- and aptamer-modified magnetic separation technique to rapidly and selectively detect *B. cereus* in food, which achieved a range of 49–49 × 10^6^ CFU/ml under optimal conditions with a detection limit of 22 CFU/ml, with a high degree of specificity. Zhou et al. (2022) established a high-resolution melting method based on a novel molecular target for discrimination between *B. cereus* and *B. thuringiensis.* The detection limit of this method could reach 1 pg of pure gDNA and 3.7 × 10^2^ CFU/ml of pure culture. Zhang et al. (2019) developed a method for rapid detection of *B. cereus* using cross-priming amplification, which achieved a detection limit of 3.6 × 10^1^ CFU/ml. A rapid, simple, accurate, sensitive, and equipment-free detection method is urgently needed for identification of *B. cereus* in emergency cases and remote resource-limited areas.

Molecular diagnostic technology is gradually replacing conventional incubation methods due to improved speed and accuracy (Ehling-Schulz et al., 2004). To efficiently differentiate among *B. cereus* group members, Park et al. (2007) established a simultaneous detection method by amplifying the *gyrB* and *groEL* genes, as diagnostic markers, using multiplex PCR in a single tube. The amplicon of the *groEL* gene was used to identify *B. cereus* group members, and the *gyrB* gene was used to further differentiate among *B. cereus* group members. With this method, the amplification step can be performed with a thermocycler within 2 h. However, the amplicons must also be separated by electrophoresis on an agarose gel for 25 min then visualized under ultraviolet light and photographed with a camera. Although efficient, this detection method still requires strenuous detection procedures and a professional operator. To shorten the time required for amplicon detection, Frentzel et al. (2016) established a method for detection of *B. cereus* based on the TaqMan qPCR assay to differentiate among *B. cereus* group members by amplifying the target genes *motB*, *bpm*, and *cry1*. The results can be obtained during amplification with a real-time fluorescence quantitative PCR instrument. The TaqMan qPCR detection method not only is more convenient but also has a lower LOD of 10^2^ CFU/ml for *B. cereus*. Hence, the major challenge for *B. cereus* is the limited use for on-site detection.

Here, the RPA-LFS assay was developed from detection of emetic and non-emetic *B. cereus*. This instrument-free assay is a promising method due to the shorter reaction time, relatively simple operation procedure, and high detection sensitivity (Li et al., 2019). The RPA-LFS assay has been applied for detection of many foodborne pathogens, such as *Campylobacter jejuni* and *Campylobacter coli*, but not *B. cereus* (Kim and Lee, 2017). Although the RPA-LFS assay can detect different pathogens on-site, the amplification products can be misidentified by the LFS (Wang et al., 2020). The exact product size can be determined by agarose gel electrophoresis or melting curve analysis, although primer–dimer formation and non-specific amplification could lead to false-negative results. When establishing the RPA-LFS assay for detection of emetic and non-emetic *B*. *cereus*, all amplicons, including the target products, non-specific products, and primer–probe dimers, could be detected by the LFS. Thus, specific strategies are needed to address this problem. For PCR or qPCR, highly specific primers and probes can reduce non-specific products and the annealing temperature could be increased to decrease the formation of primer–dimers. However, these strategies are not applicable to the highly sensitive RPA-LFS assay. Any amplicons produced by RPA and labeled with biotin or FITC could be captured by LFS, which is coated with antibodies against biotin (streptavidin) and FITC, thereby significantly increasing the risk of false-positive results.

Specific probes introduced into the RPA assay improved the specificity of the reaction (Piepenburg et al., 2006). In the present study, primers and probes were designed in strict accordance with the guidelines of the TwistAmp^®^ DNA Amplification nfo Kit. The *gyrB* gene was used as a biomarker of *B. cereus*, and the *ces* gene was used as a biomarker of emetic *B. cereus* to differentiate among *B. cereus* group members (Fricker et al., 2007; Wehrle et al., 2009; Dzieciol et al., 2012; Zhang et al., 2014; Nguyen and Tallent, 2019). The RPA amplification results were verified by traditional agarose gel electrophoresis. Then, the 5′ ends of the reverse primers and probes were labeled with biotin and FITC and the RPA products were further verified by LFS. Specific probes for *gyrB*-P combined with *gyrB*-F and *gyrB*-R were used to verify the sensitivity of the RPA-LFS assay. The expected target bands appeared at the test lines, and the NTC did not produce a positive signal. However, for detection of emetic *B. cereus*, the theoretically screened primers and probes targeting the *ces* gene produced positive signals for the NTC. Several possible reverse primer–probe dimers were evaluated. Unlike traditionally designed primers and probes, we made full use of the compatibility mismatch capability of the RPA reaction to modify the primers and probes (Daher et al., 2015; Liu et al., 2019; Wang et al., 2020). Several base substitutions were introduced, and no primer-probe dimers were produced with the use of the revised *ces*-P-m and *ces*-R-m for detection of emetic *B. cereus*.

The LOD of the established RPA-LFS assay was 10^4^ copies/ml or 10^2^ CFU/ml. For detection of contaminates at lower levels, milk samples were spiked with 10^0^–10^2^ CFU/ml of emetic and non-emetic *B*. *cereus*. The RPA-LFS assay was able to detect *B*. *cereus* at 10^2^ CFU/ml in spiked samples without enrichment or 10 CFU/ml after enrichment for 2 h. In addition, the standard culturing method was able to detect concentrations as low as 10 CFU/ml in samples without enrichment or 1 CFU/ml after enrichment for 2 h. Although standard detection methods are time-consuming, the sensitivity of the RPA-LFS assay was improved by 10-fold. After enrichment for 4 h at 30°C, the assay performed well with different spiked concentrations and cell counts were increased up to 8.7 × 10^2^–3.8 × 10^5^ CFU/ml, which was greater than the LOD of RPA-LFS. Considering the detection time and sensitivity, the performance of the RPA-LFS assay was improved for detection of higher contamination levels. Cream has high protein content and is, thus, easily contaminated by *B. cereus*. During food processing, *B. cereus* spores can contaminate food samples even at low temperatures. Therefore, to evaluate the ability of the RPA-LFS assay to detect spores in spiked food, natural contamination was mimicked by spiking cream with spores of either emetic or non-emetic *B*. *cereus* at concentrations of 10^3^–10^5^ CFU/ml and then storing the contaminated products for 2 weeks at 4°C. The RPA-LFS assay effectively detected the spores and produced results consistent with standard culture methods. Overall, the established RPA-LFS assay was able to detect *B. cereus* in artificially spiked food samples and could accurately differentiate emetic and non-emetic *B*. *cereus*.

## Conclusion

In this study, a novel method was established for detection of emetic and non-emetic *B. cereus* in dairy products by combining RPA with LFS. Colored signals were observed semiquantitatively with the naked eye on the LFS, thereby avoiding dependency on equipment and trained personnel. The developed RPA-LFS assay was able to detect emetic and non-emetic *B*. *cereus* within 20 min at an isothermal temperature of 37°C. The sensitivity of the assay was verified by detection of several common *B. cereus* group members and other foodborne bacteria. The LOD was 10^4^ copies/ml or 10^2^ CFU/ml (equal to 10 copies/reaction or 2 CFU/reaction) in pure *B. cereus* or with other bacterial strains or raw milk. Spiked cream samples were evaluated by comparing the detection results with standard culturing methods. The established RPA-LFS assay for emetic and non-emetic *B. cereus* in dairy products is rapid, simple, sensitive, and instrument-free, thereby facilitating on-site detection of emetic and non-emetic *B*. *cereus*.

## Data Availability Statement

The original contributions presented in the study are included in the article/Supplementary Material, further inquiries can be directed to the corresponding authors.

## Author Contributions

LL, WL, and YW designed the research. LW, HuY, MZ, WZ, and KW conducted the research. HaY and YS analyzed the data. LW and JD wrote the manuscript.YW directed the project. All authors contributed to the article and approved the submitted version.

## Conflict of Interest

The authors declare that the research was conducted in the absence of any commercial or financial relationships that could be construed as a potential conflict of interest.

## Publisher’s Note

All claims expressed in this article are solely those of the authors and do not necessarily represent those of their affiliated organizations, or those of the publisher, the editors and the reviewers. Any product that may be evaluated in this article, or claim that may be made by its manufacturer, is not guaranteed or endorsed by the publisher.

## Supplementary Material

Supplementary Figure 1 Specific and mismatched reverse primer/probes targeting the *ces* gene and performance in identifying emetic *B. cereus*. **(A)** Performance of *ces*-F/R/P using RPA-agarose gel. **(B)** Performance of *ces*-F/R-m/P-m using the RPA-agarose gel assay. **(C)** Exclusivity testing of the RPA-agarose gel assay for detection of emetic *B. cereus*. NTC, no template control.
