# Supplementary figures and images for: A Visualized Isothermal Amplification Method for Rapid and Specific Detection of Emetic and Non-emetic Bacillus cereus in Dairy Products
Source: Front Microbiol. 2022 Mar 28;13:802656. doi: 10.3389/fmicb.2022.802656 (PMC8996228; doi:10.3389/fmicb.2022.802656)

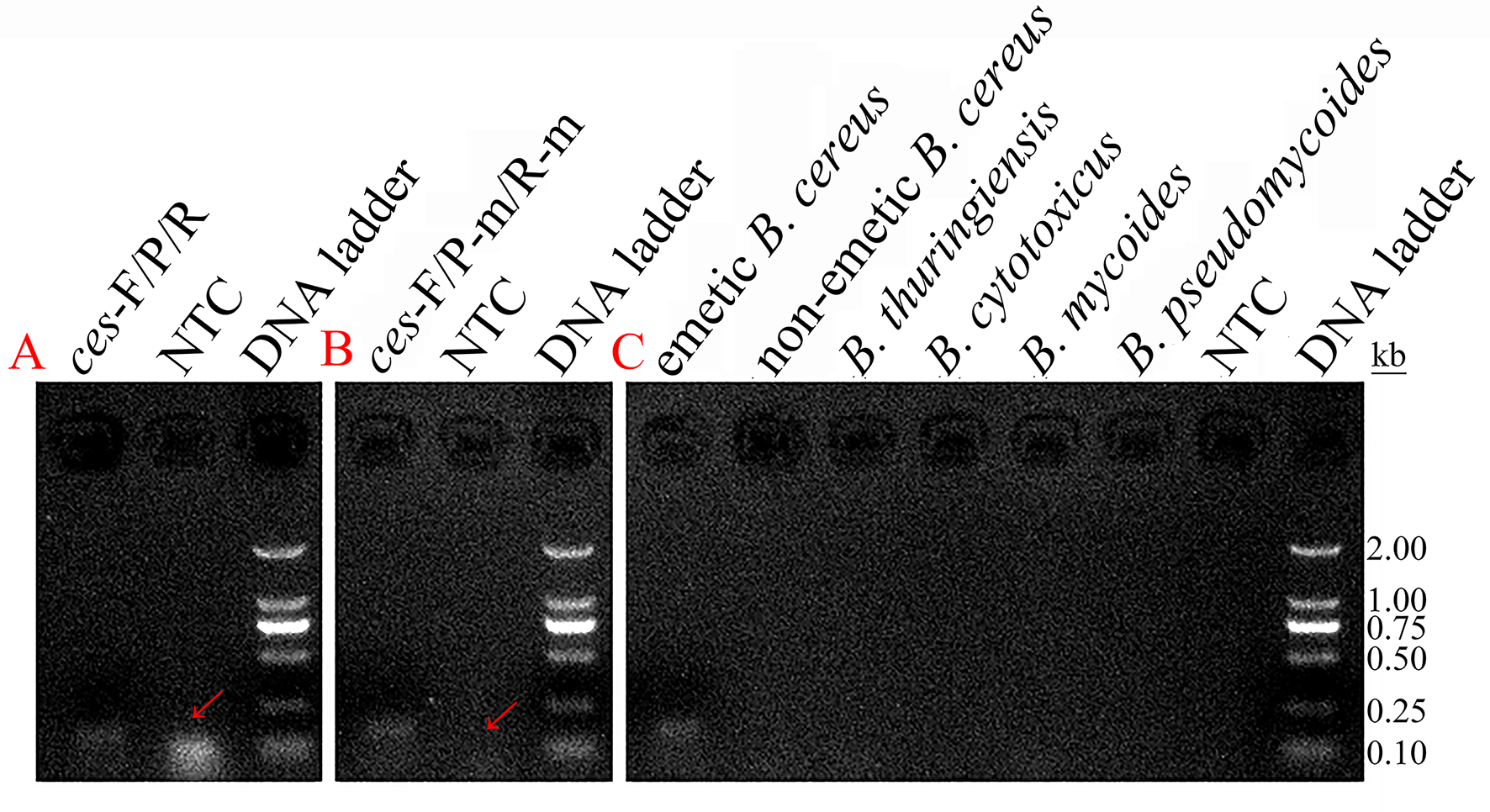

Supplement: Supplementary Figure 1 — Specific and mismatched reverse primer/probes targeting the ces gene and performance in identifying emetic B. cereus. (A) Performance of ces-F/R/P using RPA-agarose gel. (B) Performance of ces-F/R-m/P-m using the RPA-agarose gel assay. (C) Exclusivity testing of the RPA-agarose gel assay for detection of emetic B. cereus. NTC, no template control. [file Image_1.TIF]
